# Utilization of the Safe Surgical Dislocation Approach of the Hip to Retrieve a Bullet from the Femoral Head

**DOI:** 10.1155/2011/160591

**Published:** 2011-12-19

**Authors:** Ruth Delaney, Maurice Albright, Gleeson Rebello

**Affiliations:** ^1^Harvard Combined Orthopaedic Residency Program, Massachussetts General Hospital, Boston, MA 02114, USA; ^2^Department of Pediatric Orthopedics, Massachusetts General Hospital for Children, Harvard Medical School, Boston, MA 02114, USA

## Abstract

Retained intra-articular missiles from low-velocity handguns can lead to mechanical arthritis, synovitis, and lead toxicity. Various surgical approaches have been described to extract such foreign bodies from the hip joint. We present the case of a 17-year-old male in which the surgical dislocation approach was utilized to retrieve a bullet from the femoral head with a good short-term outcome. This case represents a rare application of the surgical hip dislocation approach for an unusual trauma.

## 1. Introduction

Surgical extraction of retained intra-articular missiles from low-velocity handguns should be performed to minimize the risk of mechanical arthritis, synovitis, and lead toxicity [1–3]. Various surgical approaches have been successfully utilized to extract retained intra-articular missiles [4, 5]. There are also multiple descriptions in the literature of arthroscopic techniques to remove foreign bodies from the hip joint [2, 6–8]. The surgeon may be faced with situations where the location of the foreign body makes arthroscopic retrieval difficult, or where the skills or the facility to perform hip arthroscopy on an urgent basis do not exist. Moreover, accompanying fractures of the femoral head may be difficult to address using an arthroscopic approach. Safe surgical dislocation of the hip joint as described by Ganz et al. [9] has been reported as a useful approach in assessing and treating proximal femoral and hip deformities such as slipped capital femoral epiphysis, Perthes disease, developmental dysplasia of the hip, osteonecrosis, and exostoses [10]. There is little reported on surgical dislocation as an approach for bullet extraction. We present a case in which the surgical dislocation approach was utilized to retrieve a 0.38 caliber bullet from the femoral head with a good short-term outcome.

## 2. Case Report

**Figure 1:**
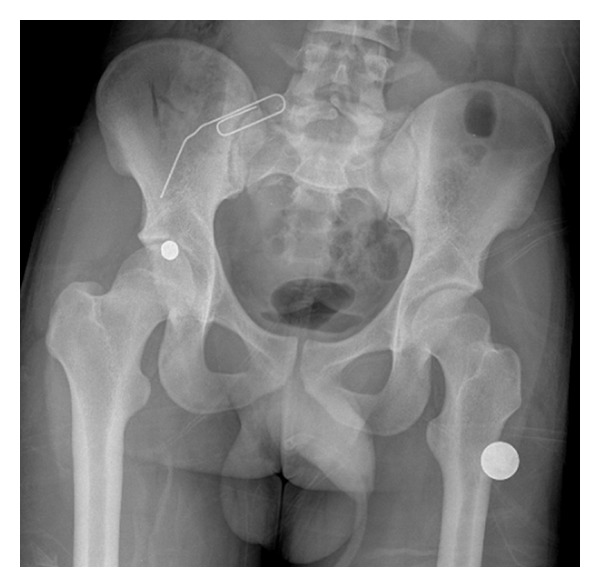
AP pelvis radiograph showing the bullet in the right femoral head. The tip of the paper clip shows the entry wound.

**Figure 2:**
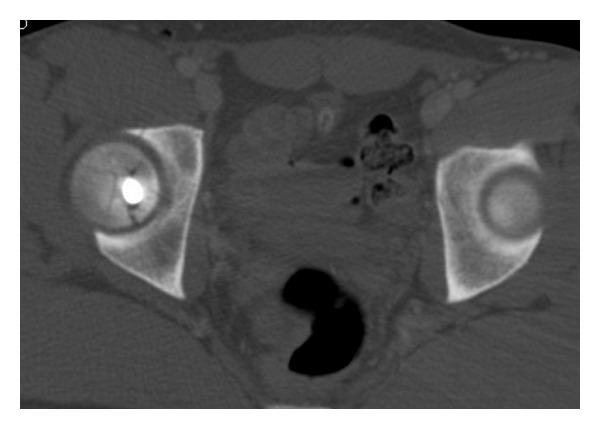
Axial CT scan of the pelvis shows the location of the bullet in the femoral head with accompanying fracture.

A 17-year-old male presented to the emergency department with a close-range gunshot wound to his right lower quadrant/groin area with an entry but no exit wound visible. He was haemodynamically stable on presentation. On initial clinical assessment in the trauma bay there was concern for an intra-abdominal injury, and emergency exploratory laparotomy was planned. A radiograph and a CT scan were performed as part of the preoperative workup. These revealed the bullet lodged in the femoral head, with associated fractures of the femoral head and the anterior lip of the acetabulum (Figures 1 and 2). Gas was seen within the soft tissues, right iliacus, and right inguinal region consistent with the bullet tract. There was no evidence of associated rupture of bowel or vasculature. The exploratory laparotomy was cancelled and orthopaedic surgery was consulted. The right lower extremity was neurovascularly intact, with no evidence of damage to the femoral neurovascular bundle. On CT imaging the femoral head fracture appeared to have multiple fragments (Figure 2). In addition, the bullet appeared embedded inside the femoral head. Hence an open approach was chosen over an arthroscopic approach. The posterior location of the bullet made access appear difficult through an anterior Smith-Peterson approach and therefore the surgical dislocation approach with trochanteric osteotomy, as described by Ganz et al. [9], was utilized.

**Figure 3:**
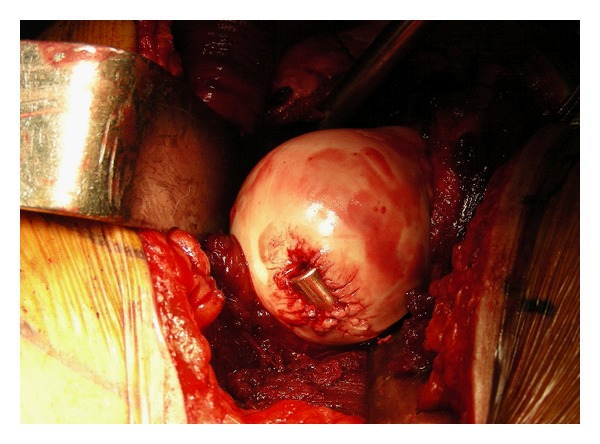
Intraoperative picture showing the presence of the bullet in the femoral head.

**Figure 4:**
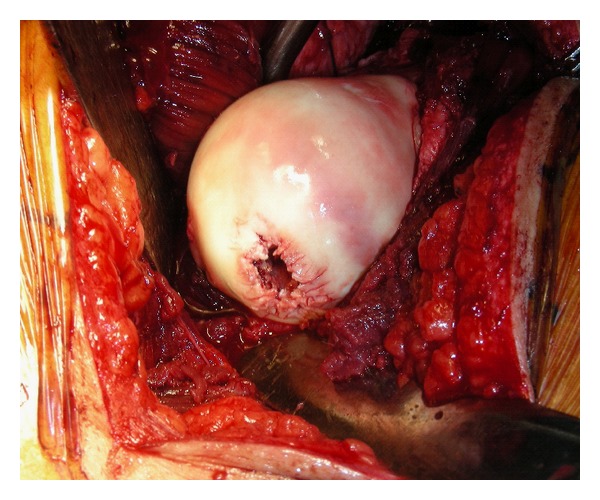
Intraoperative picture shows defect in femoral head after removal of bullet, prior to trimming and microfracture.

Upon dislocation of the femoral head, the embedded bullet was noted to be in the posterosuperior region of the femoral head (Figure 3). Other than the entry point of the bullet, there was no visible fragmentation of the femoral head. The bullet was carefully extracted using a narrow osteotome and the joint was thoroughly irrigated. Consideration was given to whether there were any osteochondral flaps that could be fixed back down to the femoral head to cover the remaining defect. However, the osteochondral fracture caused by the bullet was comminuted with no significant fragment large enough for fixation (Figure 4). The defect was curetted out, edges trimmed and microfracture was performed by drilling the base of the defect. Bone graft was not used in the defect, as the concern was that the pieces of graft would dislodge and behave as loose bodies in the hip joint.

The entry point of the bullet into the acetabulum was also visualized, with destruction of the articular cartilage in an area measuring approximately 1.5 cm^2^ in the acetabular roof, which was also microfractured. After copious irrigation with at least 6L of normal saline, the hip was reduced. Complete removal of the foreign body was confirmed by radiographs before closure. The capsule was repaired and the greater trochanter reattached using two 3.5 mm fully threaded screws. The wound was closed in layers over a drain.

The patient was comfortable postoperatively and was mobilized toe-touch weight bearing on crutches. He was restricted from any adduction, external rotation, and active abduction for 6 weeks. At two weeks postoperatively he had minimal hip pain. At his 6-week clinic visit, he had almost full range of motion of his hip and no pain. He was progressed to full weight bearing at 12 weeks postoperatively. At the end of a 18-month followup he is pain-free.

## 3. Discussion

There are a number of reports in the literature of various approaches to removing a foreign body or retained missile from the hip joint [2, 4, 5]. Arthroscopic methods, from the lateral or the inferomedial approach, have been described [6–8]. An open approach rather than arthroscopic was chosen to extract the bullet in this case because on CT imaging the femoral head fracture appeared to have multiple fragments that could potentially require fixation, and because the bullet appeared embedded inside the posterior aspect of the femoral head. We predicted difficulty with access through an anterior Smith-Peterson approach because of the posterior position of the embedded bullet on CT. Therefore the surgical dislocation approach described by Ganz et al. [9] was utilized. Safe surgical dislocation of the hip allows complete visualization of the femoral head and good access to the acetabulum. Intra-articular surgery can be performed safely, without the limitations inherent to arthrotomy without dislocation.

To the best of our knowledge, there is no described report in the literature of use of the surgical dislocation approach to extract a bullet from the femoral head. Using this approach we were able to easily visualize the bullet embedded in the femoral head, remove it safely, and perform microfracture of the femoral head defect. We believe that it would have been very difficult to achieve the same result arthroscopically or through an arthrotomy without dislocation.

The patient recovered well enough to leave the hospital on postoperative day two and had no pain with almost full range of motion of his hip at 6 weeks. However, the prognosis for his hip in the long term, having suffered a significant insult to the articular surfaces of both the femoral head and the acetabulum, is guarded.

In conclusion, surgical dislocation of the hip is a safe and effective approach for extracting a retained intra-articular missile from the hip joint, particularly when the location of the foreign body and the associated femoral head injury make other approaches such as arthroscopy or arthrotomy without dislocation less feasible.
